# Recurrent ventricular tachycardia in a patient with A19D mutation-associated hereditary transthyretin amyloidosis: a case report

**DOI:** 10.1093/ehjcr/ytae273

**Published:** 2024-06-11

**Authors:** Tanguy Bois, K Charlotte Lee, Guillaume L’Official, Erwan Donal

**Affiliations:** Department of Cardiology, Centre Hospitalier Universitaire Rennes, Pontchaillou Hospital, 2 rue Henri le Guillloux, Rennes 35000, France; University of Pennsylvania Perelman School of Medicine, Philadelphia, PA, USA; Department of Cardiology, Centre Hospitalier Universitaire Rennes, Pontchaillou Hospital, 2 rue Henri le Guillloux, Rennes 35000, France; Department of Cardiology, Centre Hospitalier Universitaire Rennes, Pontchaillou Hospital, 2 rue Henri le Guillloux, Rennes 35000, France

**Keywords:** Transthyretin amyloidosis, Hereditary, CARDIOMYOPATHY, Ventricular tachycardia, Case report

## Abstract

**Background:**

Previous literature suggests that patients with transthyretin amyloidosis (ATTR) experience a high burden of ventricular arrhythmias. Despite this evidence, optimal strategies for arrhythmia prevention and treatment remain subject to debate.

**Case summary:**

We report the case of a patient with hereditary ATTR cardiomyopathy who developed recurrent ventricular tachycardia prior to a decline in his left ventricular ejection fraction (LVEF). Although he ultimately received an intracardiac device (ICD) for secondary prevention of ventricular tachycardia, his clinical course begets the question of whether more aggressive arrhythmia prevention upfront could have prevented his global functional decline.

**Discussion:**

Given the advent of new disease-modifying therapies for ATTR, it is imperative to reconsider antiarrhythmic strategies in these patients. New decision tools are needed to decide what additional parameters (beyond LVEF ≤ 35%) may warrant ICD placement for primary prevention of ventricular arrhythmias in these patients.

**Learning points**

- Transthyretin amyloidosis (ATTR) cardiomyopathy causes fibril deposition within the cardiac conduction system, cardiac microvasculature, and myocardial tissue, provoking the development of scar tissue. This late evolution of the disease might be associated with an increased risk for ventricular arrhythmias.
- Left ventricular ejection fraction ≤ 35% is a threshold reached very late in ATTR cardiomyopathy; thus, it is imperative to elucidate other indices (e.g. left ventricular global longitudinal strain on echocardiography or scar burden on cardiac magnetic resonance imaging), which may be more useful for identifying arrhythmia risk.

## Introduction

Transthyretin amyloidosis (ATTR) is a chronic condition caused by the misfolding of transthyretin monomers into fibrils, which most commonly accumulate in cardiac and/or peripheral nervous system (PNS) tissues. Among the two forms of ATTR, wild-type ATTR (wtATTR) is the most common and primarily affects the elderly (mean age 75 years at diagnosis). Although less common, hereditary ATTR (hATTR) affects younger patients (ages 30–80 years) and is caused by transthyretin gene mutations.^1^

The clinical presentation of hATTR depends on the causal mutation involved, of which over 130 variations have been identified.^2^ Transthyretin amyloidosis–related cardiomyopathy (ATTR-CM) and PNS involvement are most common; however, ocular, gastrointestinal, and musculoskeletal manifestations can also occur, including carpal tunnel syndrome and lumbar spinal stenosis.^3^

From a cardiac standpoint, the deposition of fibrils into the myocardium causes left ventricular hypertrophy (LVH), which often precipitates restrictive cardiomyopathy and progressive heart failure. Moreover, patients with ATTR-CM may suffer from both conduction abnormalities and arrhythmias. Proposed mechanisms include the deposition of fibrils within the cardiac conduction system, inflammation, fibrosis of damaged cardiomyocytes, and myocardial ischaemia caused by microvascular fibril deposition.^4^

In addition to fluid and arrhythmia management, current strategies for treating ATTR include transthyretin stabilizers (Tafamidis) and gene silencers (patisiran and inotersen), which may slow disease progression.^5^ Clinical trials investigating monoclonal antibody treatments are ongoing.^6^ To date, the only curative treatment is orthotopic heart transplant (OHT) (± liver transplant), which is limited by the availability of donor organs.^7^ Without OHT or specific treatment, life expectancy following hATTR-CM diagnosis is 69 months.^8^

## Summary figure

**Figure:**
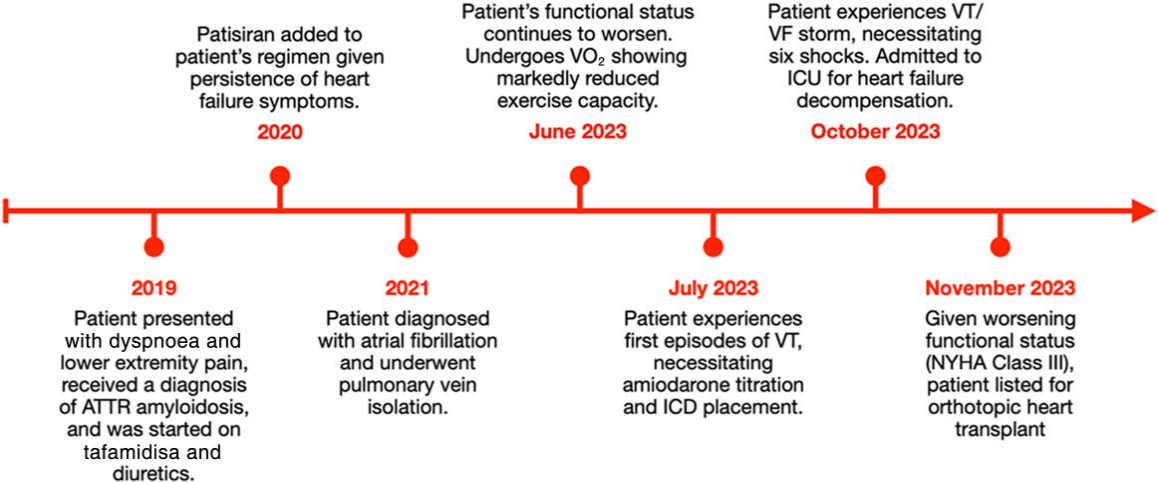


## Case presentation

**Figure 1:**
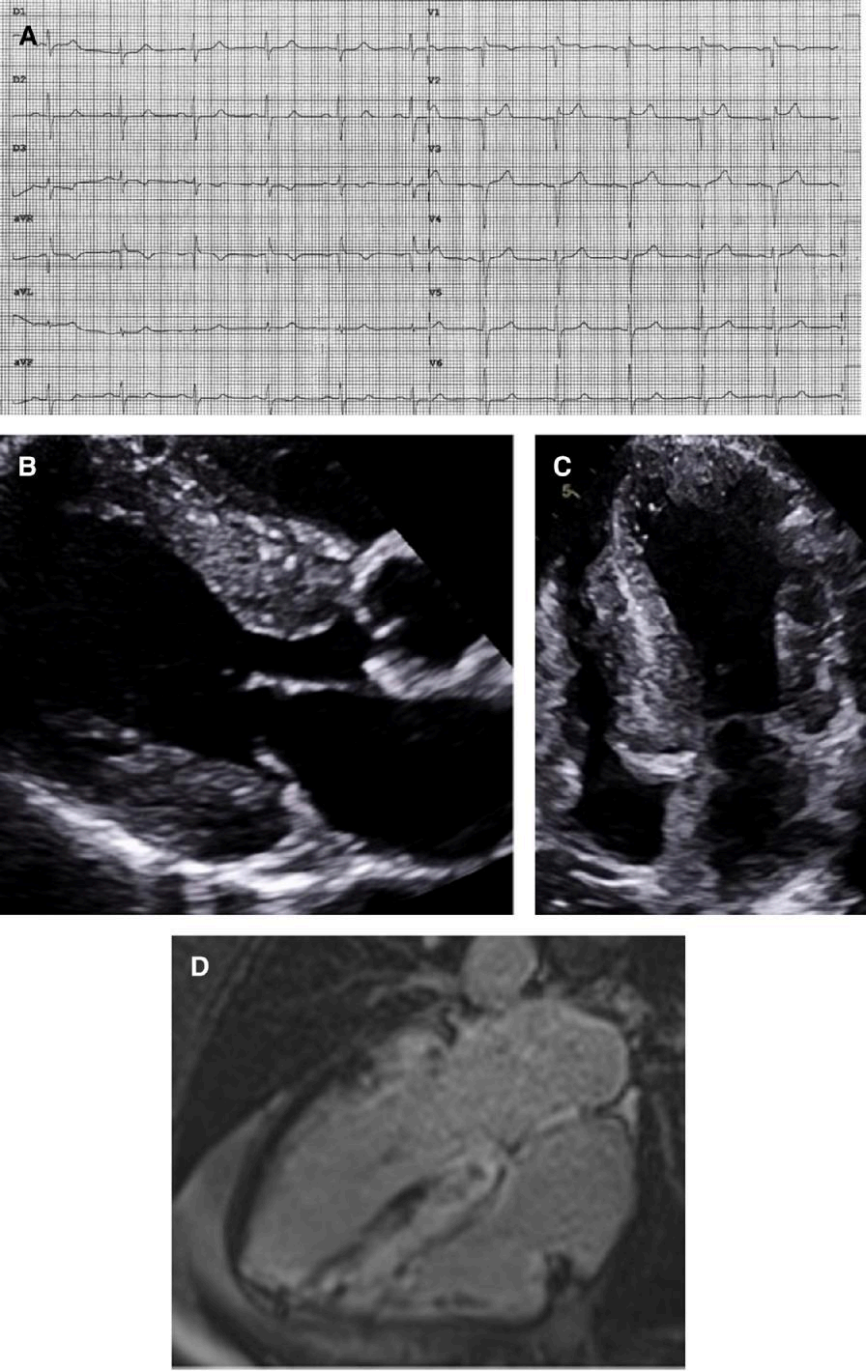
Baseline imaging utilized to establish the patient’s diagnosis of transthyretin amyloidosis–related cardiomyopathy (2019). (*A*) Baseline patient electrocardiogram with no abnormalities besides R wave flattening in V3. (*B*, *C*) Baseline patient transthoracic echocardiography, which revealed LVEF 52 with biventricular hypertrophy (septal thickness 18 mm) and left atrial dilatation (volume = 53 mL/m^2^). (*D*) Baseline patient MRI revealing fibrosis across all four cardiac chambers.

In 2019, a 57-year-old Caucasian patient with no significant past medical history was seen in our centre for dyspnoea and lower extremity pain. He underwent transthoracic echocardiography (TTE) revealing severe, concentric LVH, left ventricular ejection fraction (LVEF) 52%, and LV strain −13% with apical sparing (*Figure 1*). Given the patient’s age, this was followed by subsequent cardiac magnetic resonance imaging (MRI) (*Figure 1*) and laboratory testing to exclude light chain (AL) amyloidosis.

After confirming a diagnosis of ATTR-CM by technetium-99 scintigraphy, the patient underwent genetic testing revealing a rare A19D mutation (substitution of alanine with asparagine at position 19).^9^ Given this hereditary aetiology, thorough ophthalmologic, gastrointestinal, and musculoskeletal examinations were performed to determine the degree of extracardiac involvement, which revealed early polyneuropathy and ophthalmologic damage. At this time, the patient was started on diuretics and Tafamidis 61 mg.

In 2020, given his ongoing dyspnoea [New York Heart Association (NYHA) Class II] and neuropathy, the patient was started on patisiran. In 2021, he was found to have atrial fibrillation during exercise and underwent pulmonary vein isolation, achieving sinus rhythm.

In summer 2023, the patient returned to clinic for worsening symptomatology, including lightheadedness on exertion and functional decline. He underwent a stress test revealing VO_2_ max 15.2 mL/min/m^2^ (59% of the theoretical value) and no sustained ventricular arrhythmias. 3 dimenstional tranthoracic echocardiography showed an LVEF of 44%.

**Figure 2:**
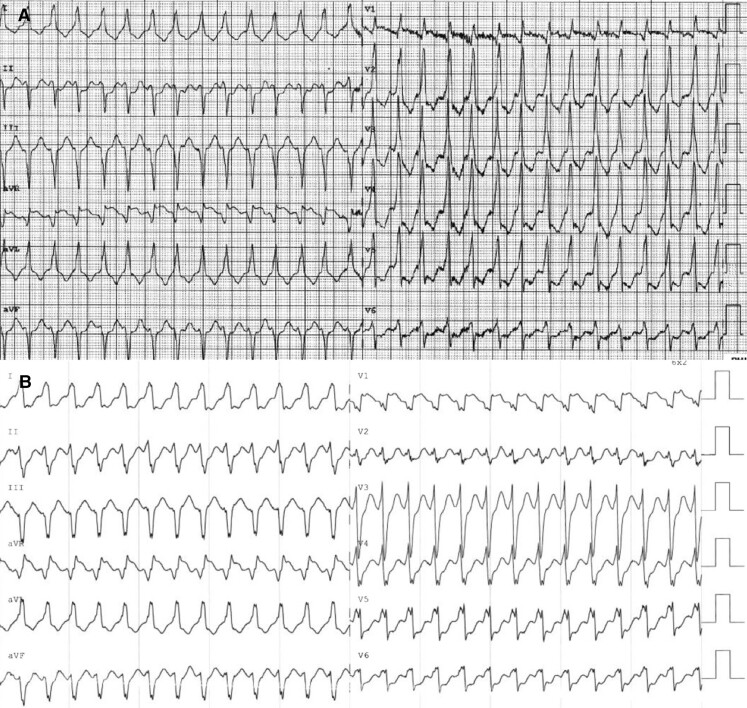
First two recorded episodes of ventricular tachycardia experienced by the patient (2023). (*A*) Patient’s first recorded episode of ventricular tachycardia (176 b.p.m.) showing a positive concordance morphology in precordial leads with a negative and inferior axis, suggesting an inferobasal septal origin. (*B*) Patient’s second recorded episode of ventricular tachycardia (162 b.p.m.) showing left bundle branch abnormality morphology, transition in V5, and superior axis, suggesting an inferomedial septal origin.

In July 2023, the patient called the local emergency medical services (EMS) for an episode of chest pain and palpitations at home. An electrocardiogram revealed a monomorphic, wide-complex tachycardia at a rate of 176 b.p.m. (*Figure 2A*), which resolved spontaneously. After a recurrence of this ventricular tachycardia (VT) in the hospital, he was started on amiodarone and admitted to the intensive care unit, where he underwent coronary angiography (without significant coronary artery lesions), repeat AL testing, and repeat TTE to exclude other causes of arrhythmia. A cardiac resynchronization therapy with defibrillator CRT-D was placed for secondary prevention of VT.

**Figure 3:**
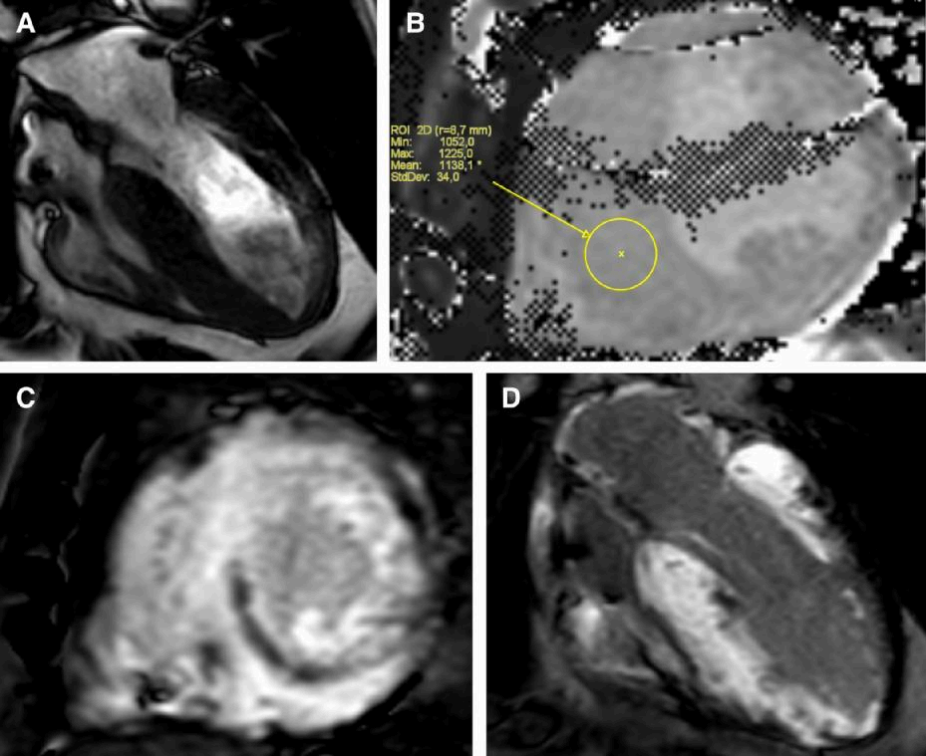
Repeat cardiac MRI performed following recurrent episodes of ventricular tachycardia (2023). (*A*) Cardiac MRI demonstrating worsening of the patient’s left ventricular hypertrophy (interventricular septum newly hypertrophied to 25 mm) and further reduction in LVEF to 26%. Notably, hypertrophy of the interatrial septum is also seen. (*B*) Elevation in the patient’s native T1 signal. (*C*, *D*) Diffuse, circumferential late gadolinium enhancement of all four cardiac chambers appreciated in 2023, which notably includes the atria and lateral wall of the right ventricle. The presence of sub-endocardial sparing argues against an ischaemic aetiology.

A few weeks later, cardiac MRI revealed a severely hypertrophied LV with diffuse fibrosis and an LVEF of 26% (*Figure 3*).

Several months later, in October 2023, the patient called the EMS again after an episode of exercise-induced VT at home. During transport, his VT deteriorated into ventricular fibrillation, necessitating six electric shocks. The only trigger found was mild hypokalaemia. He was started on bisoprolol (2.5 mg/day); however, his arrhythmias persisted (*Figure 2B*). Ventricular tachycardia ablation was considered; however, it was not pursued given the multi-focal origin of his VT.

**Figure 4:**
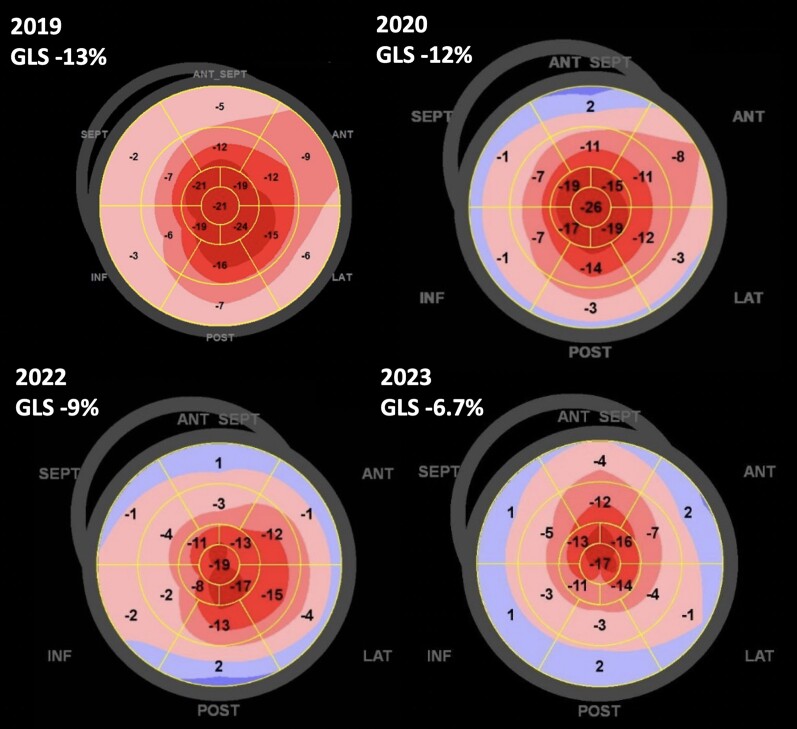
Decline in patient’s left ventricular strain from diagnosis (2019) to case conclusion (2023). Figure shows ‘bulls eye’ strain diagrams as viewed over the progression of the patient’s disease from 2019 to 2023. Dark red indicates higher left ventricular strain value; light blue indicates lower (worse) left ventricular strain.

With the progression of the patient’s symptoms (NYHA Class III) and global functional decline, he was listed for OHT and liver transplant (*Figure 4*).

## Discussion

Current literature on amyloidosis-associated ventricular arrhythmias exists; however, most studies have focused on AL amyloidosis, leaving ATTR-CM largely understudied in terms of optimal treatment strategies.

Despite this lack of guidelines, there is evidence that the burden of new VT in patients with ATTR-CM is significant. In an American cohort of 16 ATTR-CM patients with intracardiac devices (ICDs) placed for primary prevention, Kim *et al*.^10^ reported an appropriate ICD therapy rate of 25%. In another cohort of 25 hATTR patients (majority Val122Ile mutations) with ICDs placed for primary prevention, Brown *et al*. reported an appropriate ICD therapy rate of 28%. However, comparing those with LVEF ≤ 35% with and without ICDs, Brown *et al*.^11^ did not reveal any significant ICD survival benefit (3.3 ± 0.5 vs. 2.8 ± 0.4 years, *P* = 0.699).

Nevertheless, this lack of demonstrated survival benefit may be related to the fact that the current guideline for ICD placement (LVEF ≤ 35%)^12^ is a threshold reached very late in ATTR-CM. As such, LVEF may not be the best predictor of future VT risk in patients with ATTR-CM. In a Parisian cohort of 45 patients with cardiac amyloidosis (*n* = 27 hATTR, 6 wtATTR, and 12 AL) and a mean LVEF of 44.2 ± 13.1%, Hamon *et al*.^13^ studied the rates of appropriate ICD therapy (84% primary prevention, with implantation according to hospital-specific criteria). In this cohort, the appropriate ICD therapy rate was 27% with a mean LVEF of 48% in patients experiencing therapy, during a follow-up period of 17 ± 13.7 months, comparable with the ICD therapy rates present in LVEF ≤ 35% cohorts.

Beyond device-related strategies, current medical strategies for preventing and treating ATTR-associated arrhythmias are hotly debated. Given their negative chronotropic and inotropic effects, which can inadvertently reduce cardiac output in a stiff ventricle, beta-blockers are not typically recommended. However, a recent study by Ioannou *et al*.^14^ showed that low-dose bisoprolol (≤2.5 mg daily) may improve prognosis in patients with LVEF < 40%. That said, the antiarrhythmic efficacy of this dose remains uncertain. Per recent papers, amiodarone may also be used.^15^

To our knowledge, there are no data on VT ablation in ATTR patients, perhaps due to multi-focal triggers for VT in ATTR. Thus, to date, OHT remains the only curative treatment for ATTR-associated arrhythmias. Orthotopic heart transplant is more frequently pursued in hATTR patients given their younger age and lack of comorbidities relative to wtATTR. In a retrospective study of eight patients with ATTR-CM transplanted between 2008 and 2017, Kristen *et al*.^7^ showed a 5-year survival of 75%, comparable with that of patients transplanted for other heart diseases. In the event of associated neuropathy, combined cardio-hepatic transplantation is often proposed, with results comparable with heart transplantation alone (75.8% survival at 5 years). Of note, OHT is contraindicated in those with severe multi-systemic involvement (gastrointestinal damage or autonomic neuropathy).^5^

## Conclusion

Although rare, ventricular arrhythmias should be considered as a complication and a marker of poor prognosis in patients with ATTR-CM. Additional studies are needed to clarify the antiarrhythmic strategy in these patients. Given the emerging treatments for patients with ATTR-CM, strategies for assessing myocardial damage should be elucidated, as ATTR disease lesions may increase the risk for both inflammation- and scar-related arrhythmias.

## Lead author biography


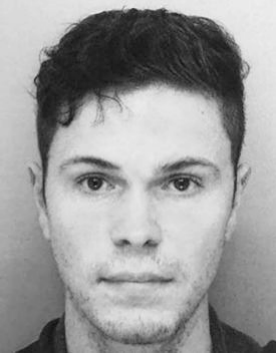


Tanguy Bois is a cardiology resident (4th year) at Rennes University Hospital, specializing in echocardiography. His fields of interest are cardiomyopathies and heart failure. He has carried out internships in specialized units in the fields of heart failure, heart transplantation, sports medicine, and intensive care unit.

**Consent:** The authors confirm that written consent for submission and publication of this case report including the images and associated text has been obtained from the patient in line with COPE guidance.

**Funding:** None declared.

## Data Availability

The data underlying this article are available in the article and in its online supplementary material.
